# Global distribution of DNA hydroxymethylation and DNA methylation in chronic lymphocytic leukemia

**DOI:** 10.1186/s13072-018-0252-7

**Published:** 2019-01-07

**Authors:** Sara Wernig-Zorc, Mukesh Pratap Yadav, Pradeep Kumar Kopparapu, Mats Bemark, Hallgerdur Lind Kristjansdottir, Per-Ola Andersson, Chandrasekhar Kanduri, Meena Kanduri

**Affiliations:** 1 Department of Medical Biochemistry and Cell Biology, Institute of Biomedicine, University of Gothenburg, Gothenburg, Sweden; 2 Department of Clinical Chemistry and Transfusion Medicine, Institute of Biomedicine, Sahlgrenska University Hospital, 413 45 Gothenburg, Sweden; 3 Department of Microbiology and Immunology, Institute of Biomedicine, University of Gothenburg, Gothenburg, Sweden; 4 Department of Internal Medicine and Clinical Nutrition, Institute of Medicine, University of Gothenburg University, Gothenburg, Sweden; 5 Department of Internal Medicine, Södra Älvsborg Hospital, Borås, Sweden

**Keywords:** Hydroxymethylation, CpG islands, Enhancers and chronic lymphocytic leukemia

## Abstract

**Background:**

Chronic lymphocytic leukemia (CLL) has been a good model system to understand the functional role of 5-methylcytosine (5-mC) in cancer progression. More recently, an oxidized form of 5-mC, 5-hydroxymethylcytosine (5-hmC) has gained lot of attention as a regulatory epigenetic modification with prognostic and diagnostic implications for several cancers. However, there is no global study exploring the role of 5-hydroxymethylcytosine (5-hmC) levels in CLL. Herein, using mass spectrometry and hMeDIP-sequencing, we analysed the dynamics of 5-hmC during B cell maturation and CLL pathogenesis.

**Results:**

We show that naïve B-cells had higher levels of 5-hmC and 5-mC compared to non-class switched and class-switched memory B-cells. We found a significant decrease in global 5-mC levels in CLL patients (*n* = 15) compared to naïve and memory B cells, with no changes detected between the CLL prognostic groups. On the other hand, global 5-hmC levels of CLL patients were similar to memory B cells and reduced compared to naïve B cells. Interestingly, 5-hmC levels were increased at regulatory regions such as gene-body, CpG island shores and shelves and 5-hmC distribution over the gene-body positively correlated with degree of transcriptional activity. Importantly, CLL samples showed aberrant 5-hmC and 5-mC pattern over gene-body compared to well-defined patterns in normal B-cells. Integrated analysis of 5-hmC and RNA-sequencing from CLL datasets identified three novel oncogenic drivers that could have potential roles in CLL development and progression.

**Conclusions:**

Thus, our study suggests that the global loss of 5-hmC, accompanied by its significant increase at the gene regulatory regions, constitute a novel hallmark of CLL pathogenesis. Our combined analysis of 5-mC and 5-hmC sequencing provided insights into the potential role of 5-hmC in modulating gene expression changes during CLL pathogenesis.

**Electronic supplementary material:**

The online version of this article (10.1186/s13072-018-0252-7) contains supplementary material, which is available to authorized users.

## Introduction

DNA methylation is a well-investigated, stable, heritable regulatory epigenetic modification in the mammalian genome and is established by interplay between maintenance DNA methyltransferase DNMT1 and de novo methyltransferases DNMT3A, and DNMT3B [1]. Evidence over the last two decades revealed that cancer DNA methylomes significantly differ from their normal tissue counter parts. Cancer genomes are characterized by global hypomethylation of the DNA, with frequent focal hypermethylation of tumor-suppressors [2, 3].

Chronic lymphocytic leukemia (CLL) has served as an ideal model system to understand the functional role of DNA methylation during cancer progression, evolution and risk stratification [4–7]. Previous studies have documented a progressive loss of methylation during B-cell development and CLL maturation [8, 9]. This progressive loss of methylation can be achieved in two alternate ways: (1) passive demethylation, by the failure of maintenance methylation following DNA replication, or (2) active demethylation, by replication-independent processes. Active demethylation is carried out by 5-methylcytosine hydroxylases TET1, TET2 and TET3 (TET refers to Ten-Eleven-Translocation); which convert 5-methylcytosine (5-mC) into 5-hydroxymethylcytosine (5-hmC) via oxidation [10, 11]. Recent studies have shown that 5-hmC is not just an intermediate product during the DNA demethylation process, but rather a stable epigenetic mark, which regulates chromatin modifications and gene transcription during developmental stages and cellular differentiation [12].

Like DNA methylation, global loss of hydroxymethylation has also been observed in many different cancers [13–16]. Importantly, loss of hydroxymethylation serves as a prognostic marker in different cancers and solid tumors [17–20]. Reduction of 5-hmC levels was also observed in certain cell types that are highly proliferative in nature and contain stem cell character such as cryptic cells of small intestine [21] and proliferative mouse neural progenitor cells [22]. Important genes regulating DNA demethylation and methylation such as TET genes, IDH genes and DNMT3A are frequently mutated in myeloid malignancies [23, 24]. Moreover, 5-hmC levels positively correlate with better overall survival [25]. In CLL, the loss of global DNA methylation levels is well documented by many researchers, including our earlier studies. Interestingly, unlike other lymphomas, CLL do not show TET mutations [26]. Hence, it would be interesting to analyze the global levels of 5-hmC in CLL and compare them with global DNA methylation levels. Also, as promoter 5-hmC has been shown to positively correlate with gene expression, it would be of interest to know whether 5-hmC plays a functional role in CLL pathogenesis via modulation of DNA methylation levels. Previous studies analysed DNA methylation changes during normal B-cell development (naïve B-cells to more differentiated memory B-cells) and CLL pathogenesis. They proposed that DNA methylation changes occurring during B-cell maturation are also recapitulated during CLL progression [27], indicating that different CLL prognostic groups derive from a continuum of maturation states reflected in normal B-cell development. Therefore, there is a need to distinguish between normal and disease-specific epigenetic events to explore the functionally important epigenetic changes that occur during disease progression.

Most studies to date have not distinguished between 5-mC and 5-hmC levels in CLL or during normal B-cell maturation, due to limitations of available methods (for example Bisulphite sequencing). Hence, it would be interesting to see how the interplay between 5-mC and 5-hmC contributes to the global levels of 5-mC, as well as gene specific changes. Towards this, we have analysed global levels and distribution of DNA methylation and hydroxymethylation during normal B-cell development and CLL maturation. To derive disease-specific epigenetic changes we have compared CLL subgroups to their corresponding cell of origin. Additionally, we have looked at the distribution of 5-hmC and 5-mC levels at both highly, poorly, and not expressed genes as well as over other regulatory regions such as enhancers, promoters and CpG islands (CGI). This combined analysis provided more insights to the potential role of hydroxymethylation in modulating progressive changes in DNA methylation landscape during CLL pathogenesis.

## Results

### Global loss of 5-hmC and 5-mC constitute a novel hallmark of CLL pathogenesis

**Fig. 1:**
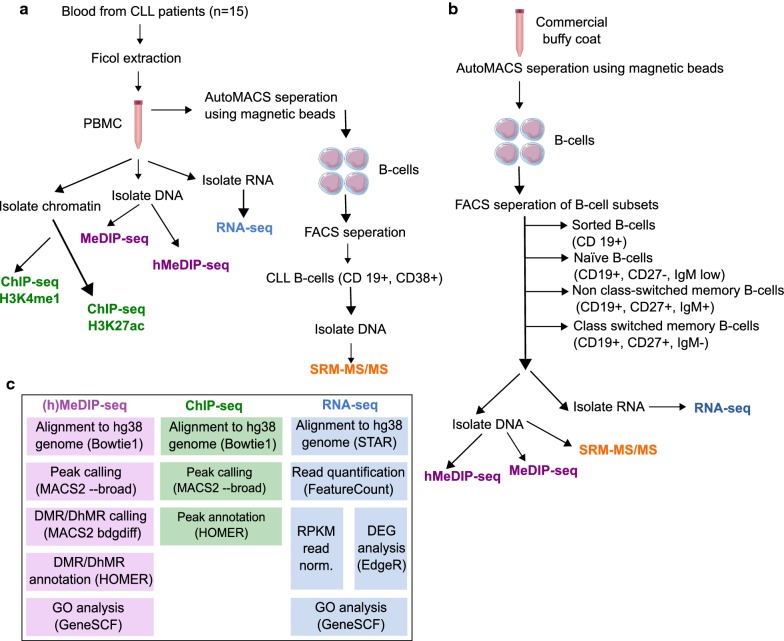
Schematic presentation of experimental design and computational analysis. Experimental design for **a** Processing CLL patient samples, **b** isolating normal B-cell and normal B-cell sub populations and **c** Pipeline used for computational analysis of sequencing data

**Fig. 2:**
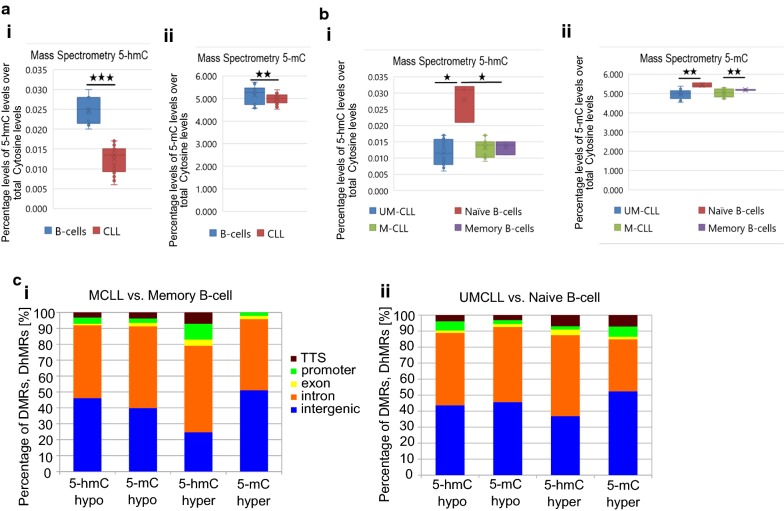
Mass spectrometry data for global 5-hmC and 5-mC levels and distribution of peaks across the genome. **a** Absolute levels of 5-hmC and 5-mC in CLL B-cells and normal B-cells, **b** absolute levels of 5-hmC and 5-mC in CLL subtypes and B-cell subsets and **c** DMR and DhMR peak distribution of uniquely mapped reads for M-CLL versus normal memory B-cell and UCLL versus normal naive B-cell comparisons across the genome

Previous studies have shown progressive changes in 5-mC levels during normal B-cell development and CLL pathogenesis, however there is no study dedicated to global 5-hmC levels. To analyse the dynamics of 5-hmC in normal B-cell differentiation and CLL pathogenesis, we employed a work-flow as described in Fig. 1. To accurately quantify 5-hmC and 5-mC global levels, we used SRM-MS on normal B-cells and CLL B-cells. We discovered a significant global decrease in 5-hmC, and 5-mC in CLL B-cells (*n* = 8) compared to normal sorted age-matched B-cells (*n* = 5), (5-hmC *p* = 0.0008; 5-mC *p* = 0.00008) (Fig. 2a). Interestingly, there was no difference in global 5-hmC nor 5-mC levels between the CLL prognostic subtypes (Fig. 2b). When all the CLL and normal B-cell sub-types were compared, we found that normal naïve B-cells showed significantly higher 5-hmC and 5-mC levels compared to other samples (Fig. 2b).

We next used immunoprecipitation-based next-generation sequencing methods (MeDIP-seq and hMeDIP-seq), with antibodies specific to 5-mC and 5-hmC, to study the global enrichment and distribution of 5-hmC levels compared to 5-mC levels (Fig. 2c). Along with normal B cells, we used naïve B-cells, NCS (non-class switched) memory B-cells, CS (class switched) memory B-cells and CLL patient samples comprising of two prognostic sub groups; IGHV mutated (M-CLL, *n* = 10) and unmutated (UM-CLL, *n* = 5) representing good and poor prognosis, respectively (Fig. 1a). Current understanding of CLL pathogenesis states that the cell of origin for M-CLL are NCS memory B-cells or CS memory B-cells and for UM-CLL the corresponding cell of origin is believed to be naïve B-cells. Hence, we compared M-CLL samples with memory B-cells and UM-CLL samples with naïve B-cells (Fig. 2c; Additional file 1: Figure S1 A–C). Yet, since the cell of origin is still debated and has not been irrefutably proven, we have also compared all CLL samples against normal B-cells. Several studies [9, 28, 29] documented global hypomethylation levels in CLL samples compared to normal healthy B-cell controls including our earlier DNA methylation study [4, 30]. Consistent with earlier results, MeDIP analysis showed fewer number of total 5-mC peaks in CLL samples compared to normal B-cells (Additional file 1: Figure S2B). Previous studies quantified 5-mC levels based on sequencing data, which has a bias toward non-repetitive regions of the genome. Since in humans, over two-thirds of the genome consists of repetitive elements, these studies saw a drastic change in 5-mC levels in cancer (Additional file 1: Figure S2B). However, absolute quantification using mass spectrometry overcome this problem (Fig. 2aii, bii). According to our MeDIP and hMeDIP analyses repetitive regions showed a lower number of total 5-hmC peaks in CLL patients, whereas unique regions showed the opposite. (Additional file 1: Figure S2B). Thus sequence-based approaches coupled with mass spectrometry-based absolute quantification method are required to make proper conclusions on global levels.

### Identification and distribution of DhMRs and DMRs across the genome

The experimental design and computational pipeline used for analyzing data and identifying differentially hydroxymethylated/methylated regions (DhMRs and DMRs) followed by functional pathway analysis is schematically shown in Fig. 1.

Distribution of CLL hyper and hypo DhMRs/DMRs in different genomic features are shown in Fig. 2ci (M-CLL vs. memory B-cell) and Fig. 2cii (UM-CLL vs. naïve B-cell). The total list of CLL hyper, hypo and common DhMRs and DMRs for all the different comparisons are listed in Additional files 3, 4: data file 2 and 3, respectively. Due to more than 90% similarity between 5-hmC and 5-mC peaks between CS and NCS memory B-cells, we used only CS memory cells in for comparisons in the rest of our study (Additional file 1: Figure S3B). Both 5-hmC and 5-mC peaks were more associated with gene-body and intergenic regions compared to promoter and transcription termination sites (TTS). However, in the M-CLL versus memory B-cell comparison, we found higher percentage of hyper 5-hmC peaks and lower percentage of hyper 5-mC peaks at promoter sites (Additional file 1: Figure S1Ai). Interestingly, in UM-CLL vs. naïve B-cell comparison, we observed lower percentage of hyper 5-hmC peaks and higher percentage of hyper 5-mC peaks at promoter sites (Additional file 1: Figure S1Aii). The difference in the percentage of hyper 5-hmC peaks, negatively correlates with percentage of 5-mC peaks at promoter sites (Additional file 1: Figure S1A), between two CLL prognostic subgroups, suggesting a dynamic role of 5-hmC modification in regulating gene promoter activity in CLL. When the percentages of total uniquely mapped 5-hmC and 5-mC peak regions including common peaks and hyper/hypo differential peaks were analysed between CLL samples and normal B-cells, we observed that the majority of 5-hmC peak regions remained unchanged (common), whereas the majority of 5-mC peak regions were hypo DMRs (Additional file 1: Figure S1B–C). As reported in Additional file 1: Figure S3A, the percentage distribution of common peaks in all the comparisons, is almost same, showing an enrichment of common 5-hmC marks equally at intronic and intergenic regions, whereas enrichment of common 5-mC marks exclusively at intergenic regions.

### Accumulation of 5-hmC over gene-body correlates with loss of 5-mC in CLL

**Fig. 3:**
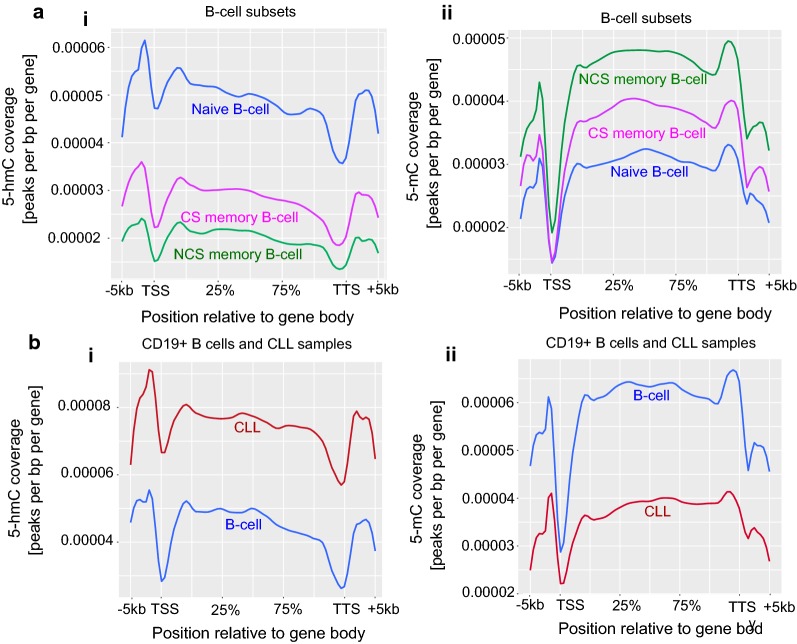
Genome-wide distribution of 5-hmC and 5-mC over the promoters and gene-body. **a** Global 5-hmC and 5-mC distribution pattern over gene-body in B-cell subsets, **b** global 5-hmC and 5-mC distribution pattern over gene-body in total CLL patient samples and normal B-cells

Enrichment of 5-hmC and 5-mC levels were plotted over gene-body across the genome, where we observed a gradual decrease in 5-hmC levels, and corresponding increase in 5-mC levels during B-cell maturation (Fig. 3a). We see more enrichment of 5-hmC across the analysed genes in naïve B-cells, compared to memory B-cells (Fig. 3ai) and the opposite for 5-mC enrichment, where naïve B-cells showed significantly lower levels of 5-mC (Fig. 3aii). Interestingly, when we compared CLL samples with normal B-cells, we found high 5-hmC levels in CLL compared to B-cells, whereas 5-mC levels showed exactly the opposite pattern, with CLL showing low 5-mC levels compared to B-cells (Fig. 3b). However, 5-hmC and 5-mC levels within CLL subgroups showed that UM-CLL had higher 5-hmC and 5-mC levels compared to M-CLL samples (Additional file 1: Figure S2C). Both normal B-cell populations and CLL samples showed similar pattern of distribution of 5-hmC and 5-mC levels across the gene-body, showing elevated levels of 5-hmC and 5-mC over the promoter-TSS regions and gene-body. However, near TTS we found a sudden depletion of 5-hmC levels and an increase in 5-mC levels in all the samples, suggesting that TTS is associated with low levels of 5-hmC and higher levels of 5-mC (Fig. 3). Taken together, these observations indicate that there is an increase of 5-hmC levels over the gene-body in normal naïve B-cells and CLL compared to normal memory B-cell and sorted B-cell samples. On the other hand, the 5-mC levels show inverse correlation to the 5-hmC levels.

### Normal distribution of 5-hmC and 5-mC over gene-body is disrupted in CLL

**Fig. 4:**
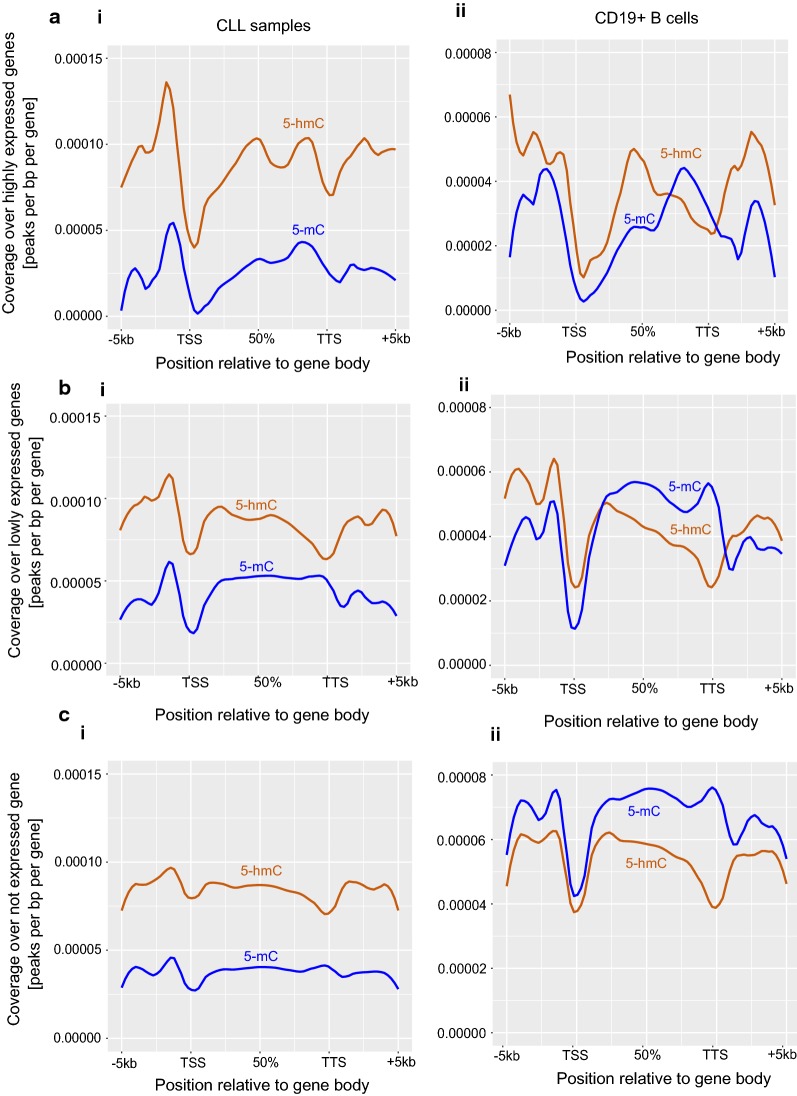
Genome-wide patterns of 5-hmC and 5-mC distribution over highly, poorly and not expressed genes. **a**–**c** shows the global 5-hmC and 5-mC distribution over gene-body of highly (**a**), poorly (**b**) and not (**c**) expressed genes. Left side plots numbered as (i) shows the data for CLL samples and the right side plots numbered with (ii) shows the data for normal B cells

Based on the expression levels of genes from RNA sequencing data of the CLL samples used in this study, genes were divided into three groups; highly expressed, poorly expressed and not expressed genes. The cut-off values and other parameters for this classification were described in the methods section. In addition to 5-hmC, and 5-mC, we have also analysed enhancer-specific marks, H3K4me1 and H3K27ac, over the gene-body of these three gene groups in all CLL samples and B-cells (Fig. 4a–c and S3D). CLL samples showed higher levels of 5-hmC compared to 5-mC over the gene-body of all three gene groups, regardless of their expression. Interestingly, at promoter regions, 5-hmC levels correlated with degree of expression among the three gene groups, where highly expressed genes were more enriched with 5-hmC (Fig. 4ai) compared to poorly expressed and not expressed genes (Fig. 4bi and c). The gradual decrease of 5-hmC levels from highly expressed genes to not expressed genes correlates with gradual decrease in H3K4me1 and H3K27ac levels over promoter regions (Additional file 1: Figure S3D). Figure 4a–dii, demonstrate enrichment of 5-hmC and 5-mC levels over the gene-body of highly, poorly and not expressed genes in normal B-cells. Unlike CLL samples, 5-hmC levels in B-cells across three gene expression groups significantly vary (Fig. 4a–dii). In highly expressed genes, there is an increase in 5-hmC levels, compared to 5-mC levels, over promoter and the 5′end of the gene-body. Interestingly, 3′end of the gene-body was enriched with higher levels of 5-mC compared to 5-hmC. This differential enrichment of 5-hmC and 5-mC across the gene-body may be crucial for proper regulation of highly expressed genes. In the case of poorly expressed genes, moderate 5-hmC enrichment was seen only at the promoters, whereas the entire gene-body region was enriched with higher levels of 5-mC (Fig. 4bii). In contrast to highly and poorly expressed genes, the not expressed genes had a significant amount of 5-mC enrichment both at the promoter and gene-body regions compared to 5-hmC and this differential enrichment was lost in CLL (Fig. 4dii). To sum up, these results clearly indicate that 5-hmC and 5-mC over highly expressed, poorly expressed and not expressed genes have defined distribution over promoter and gene-body regions in normal B-cells and this distribution is disturbed in CLL.

### Distribution and localization of 5-hmC and 5-mC levels over CpG islands, enhancers, super enhancers and promoters in CLL samples

**Fig. 5:**
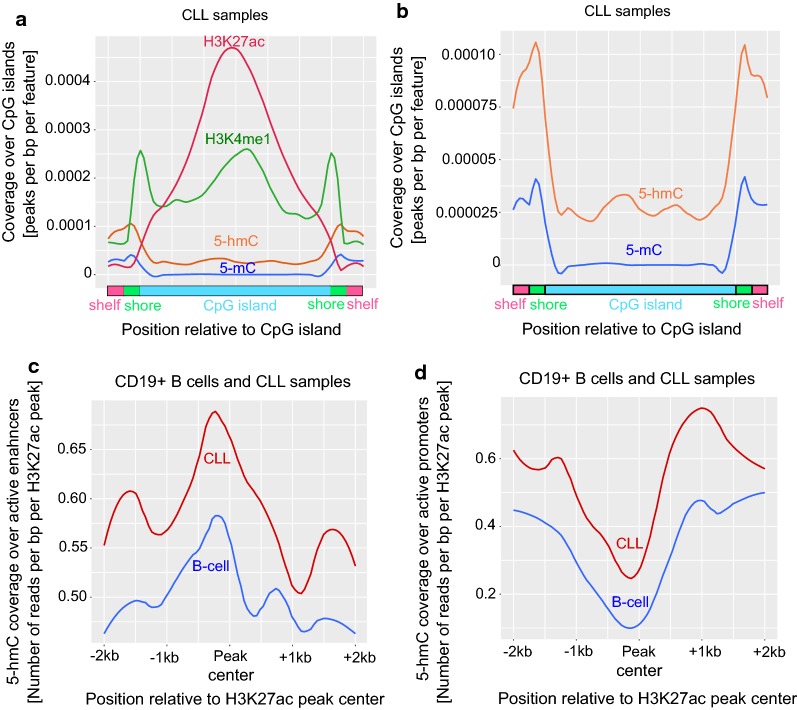
Genome-wide distribution of 5-hmC and 5-mC over CGIs, shores, shelfs, enhancers and promoters. **a** Global levels of 5-hmC, 5-mC, H3K4me1 and H3K27ac coverage over CGIs, shores and shelfs in CLL samples. **b** Global levels of 5-hmC and 5-mC coverage over CGIs, shores and shelfs in CLL samples. **c**, **d** Global levels of 5-hmC over H3K27ac peaks at active enhancers (**c**) and active promoters (**d**) in B-cells and CLL patient samples. Coverage is presented as normalized number of unique reads per bp per peak

Consistent with previous studies [31, 32] on malignant lung and liver cancers, the CGIs showed lower levels of 5-mC and relatively higher levels of 5-hmC in CLL samples. On the other hand, more 5-hmC enrichment was found at CGI shores compared to CGI. Interestingly and in line with previous data [31], CGI shores also contain higher levels of enhancer-specific mark H3K4me1 (Fig. 5a, b). Overall, 5-hmC levels were highly enriched at CGI shores, and H3K4me1 (active enhancer mark) was enriched in both CGI and CGI shores, with no enrichment at CGI shelfs. Unlike H3K4me1, H3K27ac (a mark for active enhancers and promoters) was highly enriched only at CGI and not in CGI shores or shelfs (Fig. 5a), with a clear correlation with H3K4me1 at CGI midpoint. We have also analyzed the enrichment of 5-hmC levels over all active enhancer and active promoter regions across the genome, classified based on H3K27ac mark in CLL samples. Active enhancers showed an increase of 5-hmC levels over the peak summit of H3K27ac, while at active promoter regions there is a decrease of 5-hmC levels at the peak summit (Fig. 5c, d), with a significant increase 5′ and 3’ end of the peak summit. The total list of active enhancers and promoters are provided in Additional file 6: data file 5. These results corroborates with previous studies indicating that the 5-hmC mark coincides with active enhancers but also overlaps with H3K27ac peak centers at those regions. Interestingly, the opposite can be seen at active promoter regions. Although 5-hmC is present at active promoters, it is significantly depleted at H3K27ac peak centres. Super-enhancers have been well defined in normal B-cells, however in this study we provided a list of super-enhancers during CLL pathogenesis in Additional file 6: data file 5, as a resource. Additional file 1: Figure S3C shows the cut-off used for separating predicted super enhancers from enhancers ranked by increasing H3K27ac signal.

### Biological pathways enriched in CLL samples and normal B-cells

Finally, we performed pathway enrichment analysis to investigate both biological and functional pathways enriched for hyper 5-hmC and hypo 5-hmC genes in CLL samples. GO-biological and functional pathways for CLL samples are shown for genes associated with hyper 5-hmC and hypo 5-hmC (Additional file 6: data file 5). In order to investigate the common pathways that are potentially deregulated by 5-hmC, 5-mC and gene expression levels, we performed pathway analysis using all DhMRs, DMRs and DEGs (differentially expressed genes) (Additional file 1: Figure S5). The list of DEGs for all the different comparisons between CLL and normal B-cells is provided in Additional file 5: data file 4. Several novel and known cancer pathways were found to be significantly enriched with either one or all three datasets. Additional file 1: Figure S5 shows the percentage of enrichment genes in these pathways for CLL DhMRs, DMRs and DEGs. In all 3 datasets, we see a significant enrichment of CLL-related genes and other related blood cancers from Network of Cancer Genes database (NCG 4.0) (Additional file 1: Figure S5), which suggests that all 3 datasets can independently differentiate the cancer type and similar tumor-related genes are deregulated in leukaemia and lymphomas.

### Functional role of 5-hmC enrichment in regulating differential gene expression of protein-coding genes in CLL

**Fig. 6:**
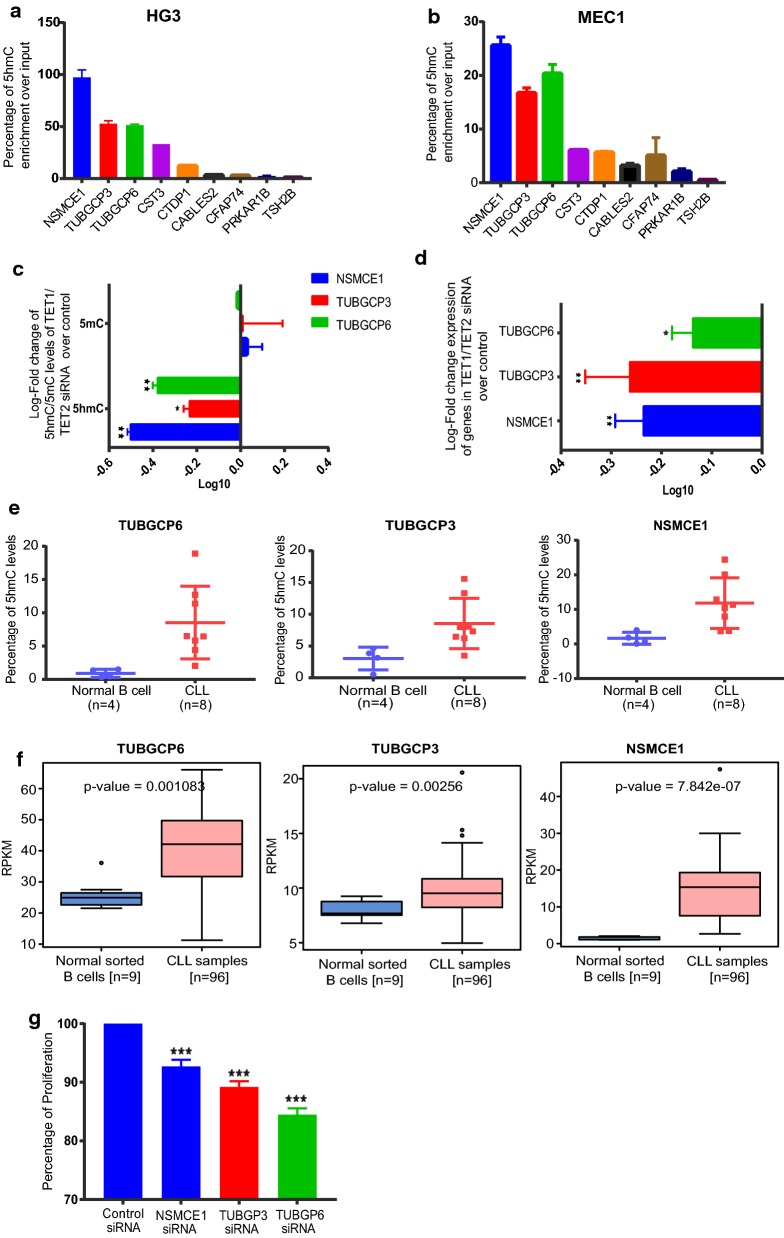
Functional relevance of 5-hmC in regulating gene expression levels. **a**, **b** 5-hmC levels of selected 5hDMR genes in HG3 and MEC1 CLL cell lines respectively. TSH2B gene was used as the negative control for hMeDIP as provided by the kit. **c** Log10-fold change of 5-hmC and 5-mC levels of HG3 TET1/TET2siRNA samples over control siRNA samples **d** Log10-fold change of relative gene expression levels over GAPDH in HG3 TET1/TET2 siRNA samples over control siRNA samples. **e** Percentage of 5-hmC levels for sorted B-CLL samples compared to normal B cell samples using quantitative epimark 5-hmC and 5-mC analysis Kit. **f** Percentage of proliferation for *NSMCE1, TUBGCP6* and *TUBGCP3* siRNA transfected HG3 samples compared to control siRNA sample using MTT assay. *Indicates *p* < 0.05, **indicates *p* < 0.005 and ***indicates *p* < 0.0005

Finally, to investigate if the alterations in 5-hmC levels of 5hDMRs results in differential gene expression levels, we selected 8 protein-coding genes from the 5hDMRs list, based on highest peak scores and their proximity to promoter and gene-body regions of genes that have functional implications in other cancers. We analyzed 5-hmC enrichment levels of these genes in two CLL cell lines; HG3 [33] and MEC1 [34] using hMeDIP analysis. Out of the 8 genes, *NSMCE1, TUBGCP6* and *TUBGCP3* showed the highest 5-hmC levels compared to the other genes in both HG3 and MEC1 cell lines (Fig. 6a, b). The expression levels of these genes in the HG3 cell line are shown in Additional file 1: Figure S4A. In order to check the role of 5-hmC levels in regulating these genes, we performed siRNA-mediated down-regulation of TET1 and TET2 genes in the HG3 cell line (Additional file 1: Figure S4B) and analysed 5-hmC and 5-mC levels using hMeDIP and MeDIP analysis on transfected samples. As shown in Fig. 6c, d, all the three genes showed significant reduction of 5-hmC levels and gene expression levels in TET1/TET2 down-regulated samples compared to control samples. However, no change in 5-mC levels (Fig. 6c) was observed. We next validated the differential enrichment of 5-hmC levels of these genes in 8 CLL (fractionated B cell samples used in SRM-MS analysis) and 4 normal B-cell samples with a quantitative-based analysis based on DNA glucosylation and restriction endonuclease digestions using the Epimark 5-hmC and 5-mC analysis Kit. All the three genes (*NSMCE1, TUBGCP6* and *TUBGCP3*) showed higher 5-hmC levels in CLL compared to normal B-cells (Fig. 6e). Of note, these observations are consistent with our global 5hmc analysis of regulatory regions. Gene expression analysis of published RNA seq data [35] (96 CLL patient samples and 9 normal B-cell samples) revealed that all three genes showed higher expression in CLL samples compared to normal B cells, supporting our findings that 5-hmC levels positively correlate with gene expression (Fig. 6f). Finally, to check the functional role of these genes in CLL progression, we performed cell proliferation assays following *NSMCE1, TUBGCP6* and *TUBGCP3* knock-down using siRNA in HG3 cell line (Additional file 1: Figure S4C). As shown in Fig. 6g, we observed a significant reduction of cell proliferation in the siRNA down-regulated HG3 cell line compared to control samples, indicating that these genes could have a potential oncogenic role in CLL.

## Discussion

Decreased global DNA methylation levels in CLL patients compared to normal healthy controls have been reported as a hallmark feature by several studies including ours [4, 9, 28–30]. However, the global levels of hydroxymethylation using more quantitative methods and its distribution across the genome has not been investigated in CLL. In this study, we provide an insight into the distribution and overall enrichment of 5-hmC and 5-mC levels in CLL samples compared to its normal counterparts using next-generation sequencing methodologies. According to our study, CLL samples exhibit lower levels of 5-hmC compared to normal CD19+ B-cells and naïve B-cells. Interestingly, we also found dynamic changes in global 5-hmC levels during normal B-cell maturation, where naïve B-cells show increased 5-hmC and 5-mC levels compared to memory B-cells, which have gone through a high proliferation phase in the germinal center. Our study indicates low 5-hmC levels might be a general property of proliferating cells, which includes malignant B-cells and cell proliferation inversely correlates with global 5-hmC levels in B-cells and CLL.

Even though 5-mC and 5-hmC have opposing functions in terms of genome regulation, their reduction in global distribution during tumorigenesis has not been well understood. Some studies have attributed global reduction of 5-hmC levels to the decreased TET enzyme activity contributed by mutations inactivating the TET enzyme activity [14, 24, 36, 37]. However, in CLL, there is no evidence of mutations affecting the TET activity. Moreover, we and others have shown that both TET1 and TET2 are expressed at higher levels in CLLB-cells compared to normal B cells [26, 38]. Despite their higher expression in CLL, like in many other cancers, 5-hmC levels are significantly low compared to normal B-cells. Studies on normal cells have concluded that terminally differentiated cells have higher global 5-hmC levels than less differentiated cells. In our study we are looking at unique cell types, B-cells, which have likely gone through higher proliferation cycles than any other cell type in a human body. Despite higher differentiation of memory B-cells than naive B-cells, we observed lower global 5-hmC levels in memory B-cells. Together with data observed by others on healthy cells and malignant cells, we propose global 5-hmC levels to anti-correlate with proliferation rate, rather than correlating with differentiation stage. Our observation that CLL genomes have higher 5-hmC levels across the gene-body and regulatory regions is consistent with its role in destabilizing the genome via creating active chromatin structures [12, 39].

A recent study on 5-hmC profiling in lung cancer showed that 5-hmC is an important epigenetic mark of active genes that is strongly associated with active histone modifications and could play an important role in gene expression mediated by DNA demethylation [31]. In line with these observations, our study also shows preferential localization of 5-hmC levels across the genome particularly at promoters, gene-body, enhancers and CpG island shores. During B-cell development, naïve B-cells showed higher 5-hmC levels compared to memory B-cells, and this 5-hmC pattern is similar to 5-mC profiles during B-cell development, where naïve B-cells showed higher CpG methylation levels compared to memory B-cells. We observed a defined 5-hmC and 5-mC patterns across the gene-body in B-cells for highly, poorly and not expressed genes. However, these defined patterns were completely lost in CLL.

Finally, our global study identifies the importance of TET-mediated 5-hmC enrichment in regulating the differential expression of three oncogenes: *NSMCE1, TUBGCP3* and *TUBGCP6*. *NSMCE1* gene was shown to play key roles in the maintenance of chromosome integrity during mitotic proliferation, meiosis, and DNA repair and is critical for genome stability [40] whereas *TUBGCP6* and *TUBGCP3* genes were shown to be over-expressed in glioblastoma [41]. Down-regulation of these genes in CLL cell lines resulted in a significant decrease in cell proliferation, which further suggest that these genes could have a role in CLL progression. According to mass spectrometry analysis, global 5hmC levels in CLL B cells are lower compared to 5mC levels. However, the functional role of 5hmC levels in the differential expression oncogenes in CLL cell lines, indicate that 5hmC even at low levels may contribute to differential gene expression. Nevertheless, more functional studies on CLL primary samples are warranted to understand the direct functional implications of 5hmC at these lower levels in CLL.

Hence, the current investigation, in addition to identifying three oncogenes genes with potential roles in CLL progression, characterize 5-hmC and 5-mC patterns underlying the aberrant gene expression in CLL.

## Methods

### Patient samples and clinical data

A total of 16 CLL patients were included in this study. The CLL peripheral blood mononuclear cell (PBMC) samples were collected from the Section of Hematology and Coagulation, Sahlgrenska University Hospital. The CLL patients were diagnosed according to recently revised criteria [42] and samples were collected at the time of diagnosis. Clinical and molecular data are summarized in Additional file 2: data file 1A. All patients provided informed consent in accordance with the Helsinki Declaration and the study was approved by the local ethics review board. Genomic DNA and total RNA were extracted from CLL PBMCs and sorted B cell subpopulations with DNA (DNeasy Blood & Tissue Kit, 69504, Qiagen, Hilden, Germany) and RNA (miRNeasy mini kit, 217004, Qiagen, Hilden, Germany) extraction kits according to manufacturer’s protocol. The quality of RNA was measured using Experion RNA analysis kit (7007103, Bio-Rad, Hercules, USA). Five age-matched sorted CD+ 19 B cell DNA and RNA were bought commercially (3H Biomedical, Uppsala, Sweden). The quality of RNA was checked using 2100 Bioanlyser Instrument (Agilent, Santa Clara, United States) and the sent for RNA sequencing.

### Isolation of normal B cell subpopulations and CLL B cells from CLL PMBC samples

Four buffy coats from normal healthy blood donors age matched with CLL patients were collected from Sahlgrenska university hospital. PBMC were isolated from the buffy coats using Lympho-Prep (lymphoprep, 1114545, Axis-shield, Oslo, Norway) density gradient sedimentation and were then enriched for B cells on a AutoMACS using CD20 microbeads in accordance with the instructions from the manufacturer (Miltenyi Biotec, Bergisch Gladbach, Germany). After separation, B lymphocytes the purity of preparations was checked by flow cytometry which showed around 96% to 98% for CD19+ cells. Then the sorted B cells were stained with BB515-labeled anti-CD19, PE-labeled anti-IgM and BV421-labeled anti-CD27 antibodies before flowcytometric cell sorting using a BD FACSAria cell sorter (BD Bioscience, San Jose, USA). B cells were sorted into naïve (CD19^+^, CD27-), memory (CD19^+^, CD27^+^, IgM^−^) and marginal-zone like (CD19^+^, CD27^+^, IgM^−^) B cell populations [43]. CLL B cell DNA used for Mass Spectrometry analysis was isolated from CLL PBMC patient samples in similar way as described for normal B cell isolation from normal PBMCs, using AutoMACS.

### Selected reaction monitoring liquid chromatography tandem-mass spectrometry (HPLC-SRM-MS)

An SRM-based mass spectrometry assay (SRM-MS) was used to quantify 5-hydroxymethyl-2′-deoxycytidine (5-hdmC) and 5-methyl-2′-deoxycytidine (5-mdC) concentrations as a percentage of 2′-deoxyguanosine (dG) (e.g.—[5hmdC]/[dG] and [5mdC]/[dG]). The calibrated ranges for the analytes were 0–2.5% for 5hmdC and 0–25% for 5mdC using a fixed 40 pmol amount of dG as an internal standard. The calibration points were run as single replicates due to previously demonstrated high reproducibility of the assay. The samples had a measured range of 5hmdC as low as 0.01% and as high as 0.028%. The samples had a measured range of 5mdC between 4.61% and 5.69%.

### MeDIP, hMeDIP and ChIP assay

MeDIP and hMeDIP assay was performed using MagMeDIP (C02010021, Diagenode, Liege, Belgium) and hMeDIP kits (C02010030, Diagenode, Liege, Belgium), respectively, according to manufacturer instructions using Mouse monoclonal antibody against 5-mC (33D3 clone, C15200081, Diagenode, Liege, Belgium) and Rat monoclonal antibody against 5-hmC (C15220001, Diagenode, Liege, Belgium). ChIP was performed using Shearing module kit and the OneDay ChIP Kit (Diagenode, Liege, Belgium), according to the manufacturer’s instructions. Briefly, genomic DNA (~ 3ug of for MeDIP and ~ 10ug for hMeDIP) was sonicated for 5 times with 30 s on and 30 s off for 4 cycles each time to obtain 300–600 bp chromatin using Bioruptor and shearing module kit (Diagenode, Liege, Belgium). 1% of fragmented DNA was removed as input sample into a fresh tube. The sheared DNA samples were incubated with magnetic beads and antibody at 4^0^ C for overnight. After overnight incubation the unbound DNA was removed from antibody- bead mix and washed three times. The DNA was extracted from the beads and purified by phenol, chloroform and isoamylalcohol method.

ChIP was performed using Shearing module kit and the OneDay ChIP Kit according to the manufacturer’s instructions. The antibodies used were polyclonal antibody against H3K4me1 (C15410037, Diagenode, Liege, Belgium), polyclonal antibody against H3K27ac (C15410174, Diagenode, Liege, Belgium) and IgG (negative control; OneDay ChIP Kit). In brief, the CLL PBMCs were formaldehyde-crosslinked, lysed, and sonicated four times for 5 cycles (each cycle 30 s on and 30 s off) with Bioruptor and the Shearing module kit (Diagenode, Liege, Belgium). The concentration of resulting DNA fragments was determined by Qubit 2.0 fluorometer (Q32866, Invitrogen, Carlsbad, USA) and sent for MeDIP ans hMeDIP sequencing perfromed using Ilumina Hiseq 2000 platfrom.

### Data processing and analysis of hMeDIP-seq, MeDIP-seq and ChIP-seq data

Adapter sequence from raw sequencing reads were removed using Cutadapt v2.2.1. Cleaned reads were than aligned to human GRCh38 reference genome, using Bowtie v1.0.0 --best -n 2 -k 1 -m 1 -t [44]. Sex chromosomes, X and Y, were removed from further analysis to exclude gender bias. Aligned reads were used to call peaks with MACS v2.1.0 -f BAM --broad --broad-cutoff 0.05 -B -g hs, over corresponding inputs. The details and summary of all the obtained reads from CLL samples and normal control samples used in this study are listed in Additional file 2: data file 1.

Aligned reads were used to call peaks with MACS v2.1.0 [45] -f BAM --broad --broad-cutoff 0.05 -B -g hs, over corresponding input samples. After peak calling for each sample, UCSC’s utility WigCorrelation was used on BED files, to estimate the correlation between samples. Since correlation was high between samples, another round of peak calling was performed, with the same parameters, this time peak calling was done simultaneously on all IGHV-mutated CLL samples, all IGHV-unmutated CLL samples and all CLL samples, regardless of IGHV mutational status, together. The details and summary of all the obtained reads from CLL samples and normal control samples used in this study are listed in Additional file 2: data file 1B. For MeDIP-seq and hMeDIP-seq an additional step was done, where CLL Differentially methylated Regions (DMRs) and CLL differentially hydroxymethylated regions (DhMRs) were analyzed, using MACS v2.1.0 bdgdiff. Comparisons were done the following way: CLL samples versus sorted B cells, IGHV-unmutated CLL samples vs. Naive B cell and IGHV-mutated CLL samples versus Memory B cell. Peak regions, DMRs and DhMRs were assigned to genes and other genomic features using HOMER v4.9 annotatePeaks, with a custom GTF annotation file from Gencode v24. GeneSCF v1.1 was used for pathway enrichment analysis of protein coding genes associated with DhMRs and DMRs, using KEGG and NCG databases and p-value 0.05 and FDR 0.1 as cut-offs. For visualization, HOMER v4.9 [46] makeMetaGeneProfile and DeepTools v2.3.1 computeMatrix and plotProfile were used. Plotting was done in R v3.2.3, using ggplot2 and reshape2. All the raw data has been deposited in GEO, with the accession number GSE113386 and will be available for download to the public after acceptance.

### Analysis of RNA-seq data

Raw reads containing adapter sequences, were removed, using CutAdapt v2.2.1. Cleaned reads were aligned to GRCh38 reference genome, using STAR v2.5.2b. Aligned reads were used for quantification, using SubRead v1.5.2 FeatureCount with Gencode v24 annotation. Normalization of read counts was performed with RPKM normalization, using an in-house script. Genes were separated in highly (RPKM 100 or more), intermediately (10–100), lowly (RPKM 1–10) and not (RPKM less than 1) expressed. Differential expression analysis was performed in R v3.2.3, using EdgeR. Comparisons were done the following way: CLL samples versus sorted B cells, IGHV-unmutated CLL samples versus naïve B cell and IGHV-mutated CLL samples versus memory B cell. GeneSCF v1.1.2 was used for pathway enrichment analysis of DE protein coding genes, using KEGG and NCG databases and *p* value 0.05 and FDR 0.1 as cut-offs. For validating the gene expression levels from CLL published RNA seq data [35], we obtained the raw data of RNA-seq samples for 96 patients (55 IGHV-mutated and 41 IGHVunmutated prognostic groups) along with 9 normal B cell samples as described in our earlier paper [30].

### Quantitative analysis of 5hmC levels

DNA glucosylation and restriction endonuclease digestions were performed using the Epimark 5-hmC and 5-mC analysis Kit (NEB, Ipswich, MA) as per the manufacturers instructions. The primer sequences used in this analysis were listed in Additional file 2: Supplementary Table 1. A total of 5ug of genomic DNA was treated with T4 β-glucosyltransferase with and without UDP-Glucose substrate at 37 °C for overnight. Glucosylated DNA was digested with and without MspI and HpaII at 37 °C for overnight. 5hmC levels were quantitatively analysed using Real time Q-PCR with primers designed at peak regions containing GGCC sequence on target genes which were shown to be differentially hydroxymethylated between CLL samples and normal B cells (Additional file 7).

### Analysis of super-enhancers

For the analysis of super-enhancers in CLL, ROSE software was used, with the following parameters: -g HG38 -i CLL-H3K27ac_peaks.gff -r f -r CLL_H3K27ac_aligned.bam -t 2500.

### Cell lines, culture conditions, siRNA transfections and MTT assay

Two CLL cell lines, HG3 and MEC1 were used in this study for functional analysis. The cell lines were cultured in RPMI 1640 (Invitrogen), Carlsbad, USA) supplemented with glutamine (2 mM glutamine), 10% fetal bovine serum (FBS; Invitrogen, Carlsbad, USA), and 1× penicillin/streptomycin (Invitrogen, Carlsbad, USA). Transient transfections were carried out using Amaxa 4D-Nucleofector™ System (Lonza group AG, Basel, Switzerland) using the SF cell line Amaxa kit (V4XC-2032) according to the manufacturer’s instruction. We used MISSION Pre-designed siRNA (Sigma Aldrich, Missouri, USA) containing five small interfering RNAs (siRNAs) in equal concentrations for NSMCE1, TUBGCP6 and TUBGCP3 genes. Predesigned Stealth siRNAs were used for TET1 and TET2 (#HSS129586; #HSS12325; ThermoFischer Scientific, Waltham, USA). The silencer negative control siRNA (ThermoFischer Scientific, Waltham, USA) was used as control siRNA. Cell proliferation was analyzed using MTT assay after 48 h of post transfection using siRNAs specific for selected target genes with control siRNA as mentioned above. The MTT assay was performed according to the manufacturer’s protocol using Cell Titer 96 Non-Radioactive Cell Proliferation assay kit (G4000, Promega Madison, USA).

## Additional files

**Additional file 1.** Additional figures 1 to 3 and the list of all additional files and contents

**Additional file 2.** Clinical and molecular data of patient samples and summary of obtained reads from global data

**Additional file 3.** List of common, hyper and hypo DhMRS between CLL samples and normal controls comparisons

**Additional file 4.** List of common, hyper and hypo DMRS between CLL samples and normal controls comparisons

**Additional file 5.** List of common, hyper and hypo DEGS between CLL samples and normal controls comparisons

**Additional file 6.** List of REACTOME and GO Biological pathways from DhMRs obtained between CLL samples and normal controls comparisons and list of CLL active and super enhancers based on H3K27Ac ChIP seq data

**Additional file 7.** Mass spectrometry data and list of primer sequences used in this study

## Abbreviations

5-hmC: 5 hydroxymethyl cytosine
5-mC: 5 methyl cytosine
CLL: chronic lymphocytic leukemia
MTT assay: 3-(4,5-dimethylthiazol-2-yl)-2,5-diphenyltetrazolium bromide assay
TET: ten-eleven-translocation
NGS: next-generation sequencing
MeDIP: methylated DNA immunoprecipitation
hMeDIP: hydroxyl methylated DNA immunoprecipitation
CGI: CpG island
DMR: differentially methylated region
DhMR: differentially hydroxymethylated region
