# Supplementary material for: Global distribution of DNA hydroxymethylation and DNA methylation in chronic lymphocytic leukemia
Source: Epigenetics Chromatin. 2019 Jan 7;12:4. doi: 10.1186/s13072-018-0252-7 (PMC6322269; doi:10.1186/s13072-018-0252-7)
Supplement: Supplementary file 1 — Additional file 1. Additional figures 1 to 3 and the list of all additional files and contents [file 13072_2018_252_MOESM1_ESM.pdf]

Fig. S1

Figure S1. 5-hmC and 5-mC distribution across the genome in CLL patients and sorted B-cells.
(A) Percentage of DhMRs and DMRs of uniquely mapped reads at promoter regions in mutated CLL vs. Memory B-cell (i) and unmutated CLL vs. Naive B-cell (ii). (B) Percentage of unchanged, hypo and hyper 5-mC levels in mutated CLL vs. Memory B-cell (i) and unmutated CLL vs. Naive B-cell. (ii). (C) Percentage peaks that fall into category of unchanged, hypo and hyper 5-hmC levels in mutated CLL vs. Memory B-cell (i) and unmutated CLL vs. Naive B-cell (ii).

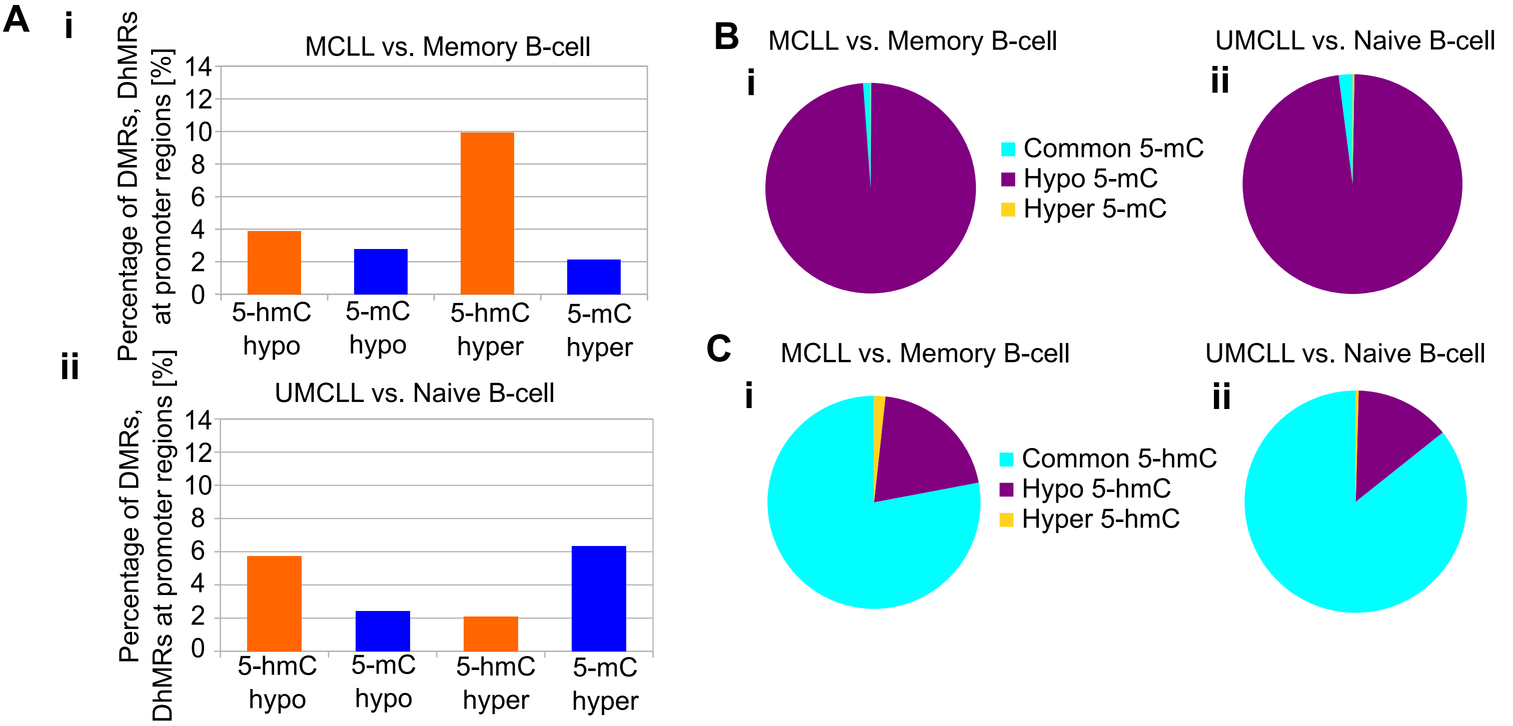

Fig. S2

Figure S**2**. Peak numbers, Repeat analysis and enrichment over gene body for 5-mC and 5-hmC.
(A) 5-mC (i) and 5-hmC (ii) enrichment of repeat regions in CLL and B-cells. (B) Total peaks detected with MACS2 in repetitive regions (i) and unique regions (ii). (C) Global 5-hmC (i) and 5mC (ii) distribution pattern over gene body in unmutated and mutated CLL subtypes.

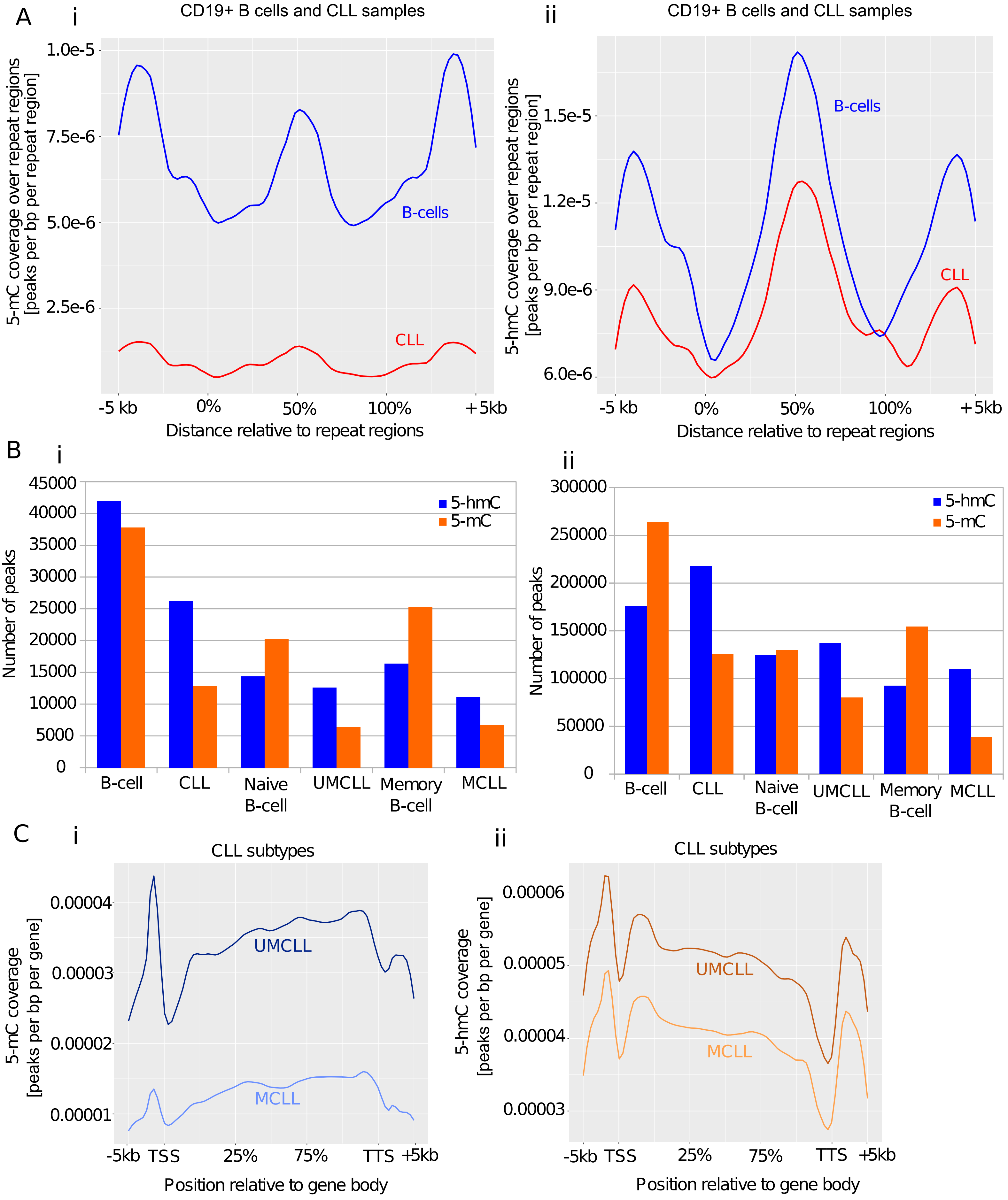

Fig. S3

Figure S . Peak distribution, super enhancer analysis and enrichment over highly/lowly/not expressed genes. (A) Peak distribution of common/unchanged 5-hmC and 5-mC marks in mutated CLL vs. Memory B-cell (I) and unmutated CLL vs. Naive B-cell (ii). (B) DMR and DhMR distribution in Memory B-cell vs. NCS memory B-cell. (C) ROSE super enhancer analysis. (D) Enrichment of H3K4me1, 5-mC and 5-hmC over gene body of highly (i), lowly (ii) and not (iii) expressed genes in CLL patient samples.

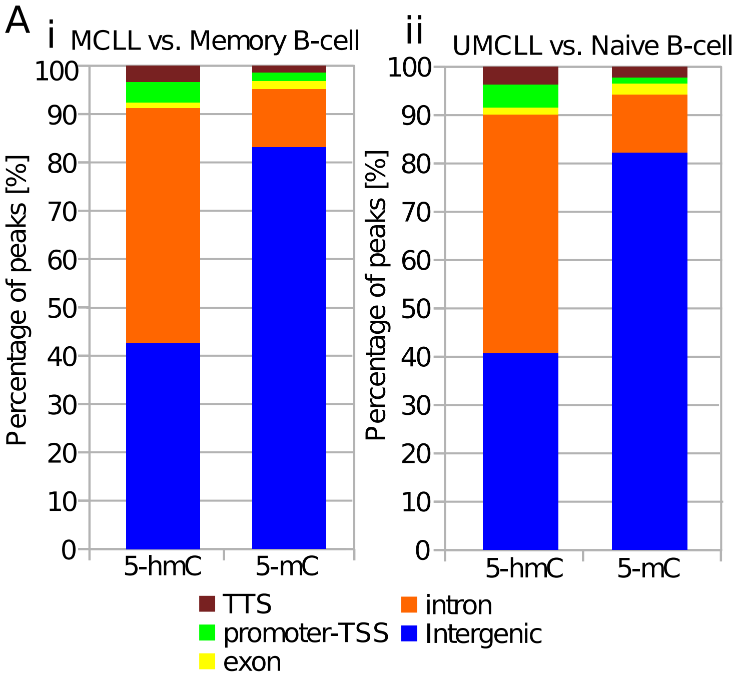

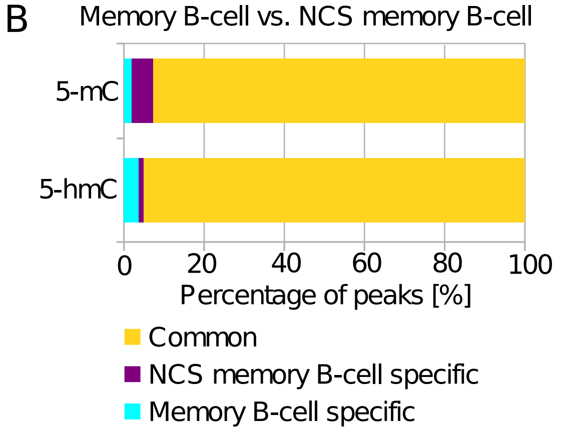

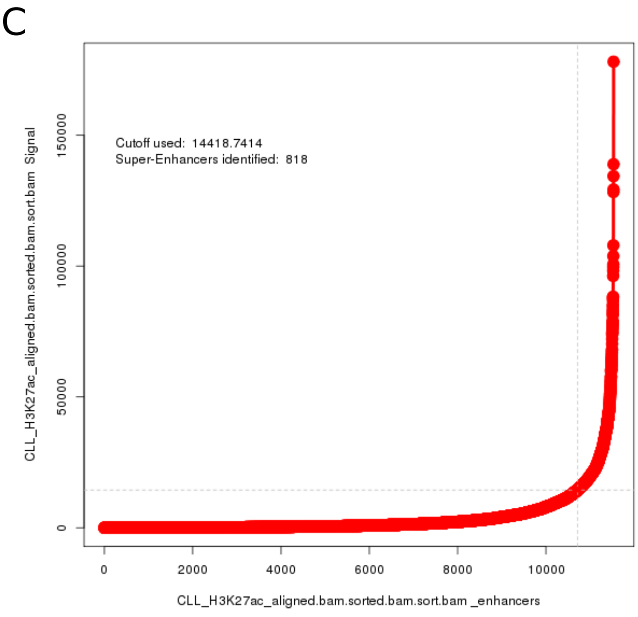

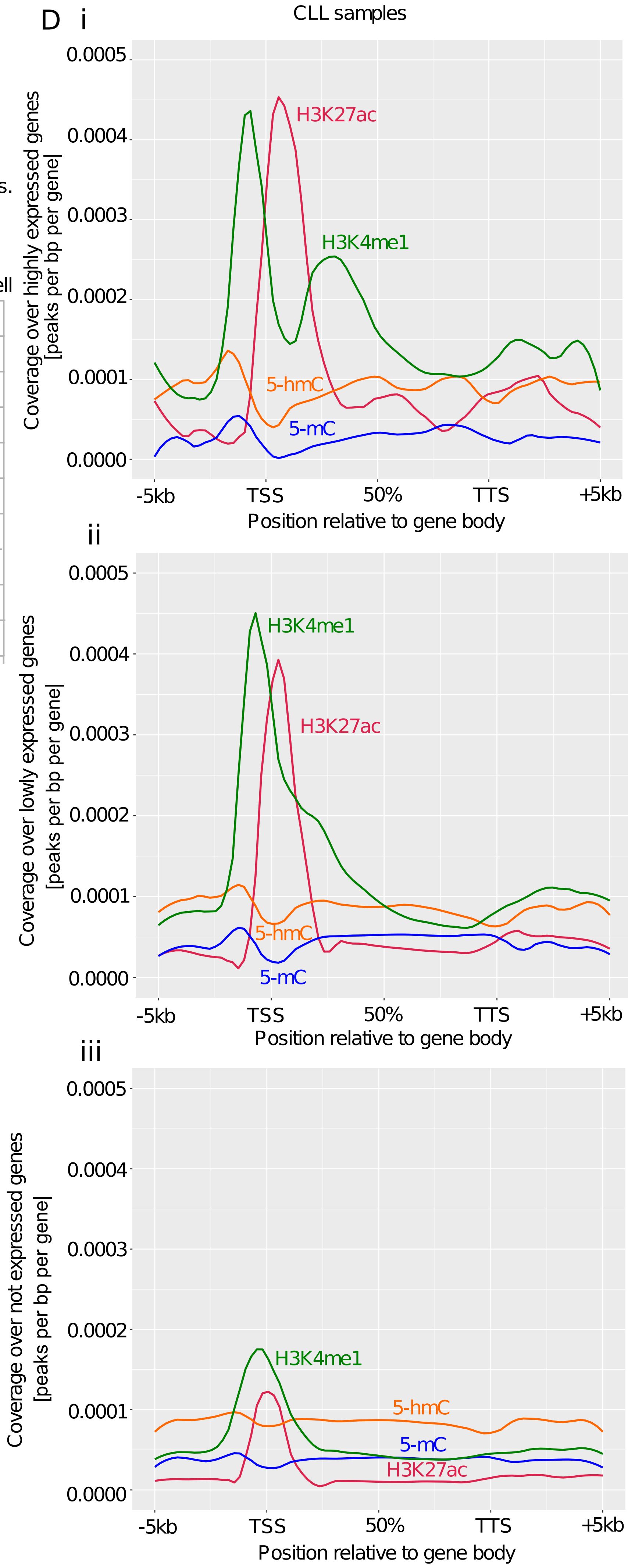

Fig. S4

Figure S4. Gene expression and 5hmC / 5mC levels in CLL cell lines

A) Expression of all the selected genes for hMeDIP analysis in HG3 cell line. D-E) Relative expression of genes after down-regulation in HG3 cell line (B- TET1 and TET2) and (C- NSMCE1, TUBGCP3a dn TUBGCP6). * indicates p<0.05 and ** indicates p<0.005.

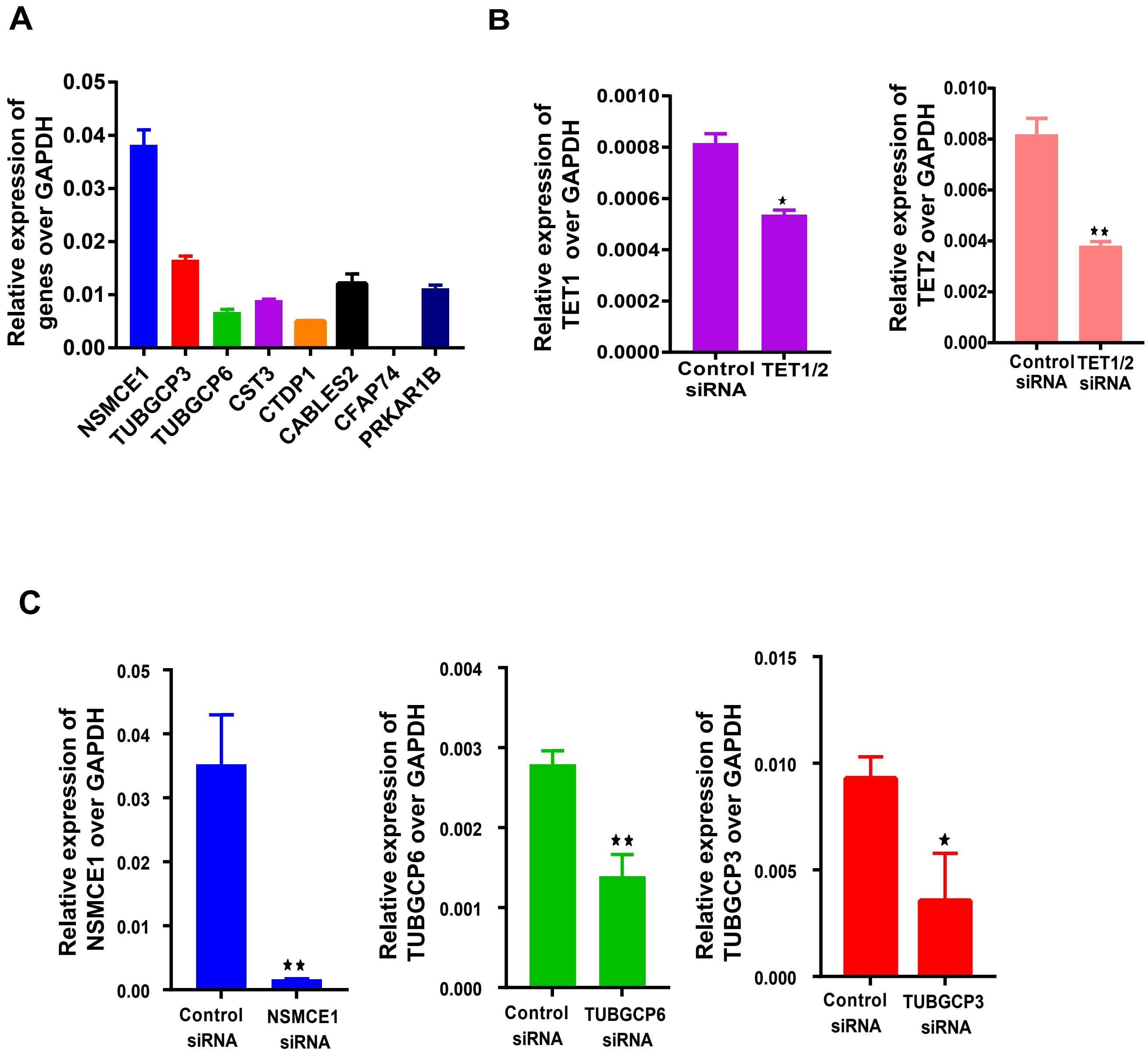

Fig. S5

Figure S5. Validation of sequencing data and enriched functional pathways.
(A) Common enriched pathways in DhMRs, DMRs and DEGs in CLL patients compared to sorted B-cells. (B) Enriched cancer pathways in DEGs, DhMRs and DMRs.

A

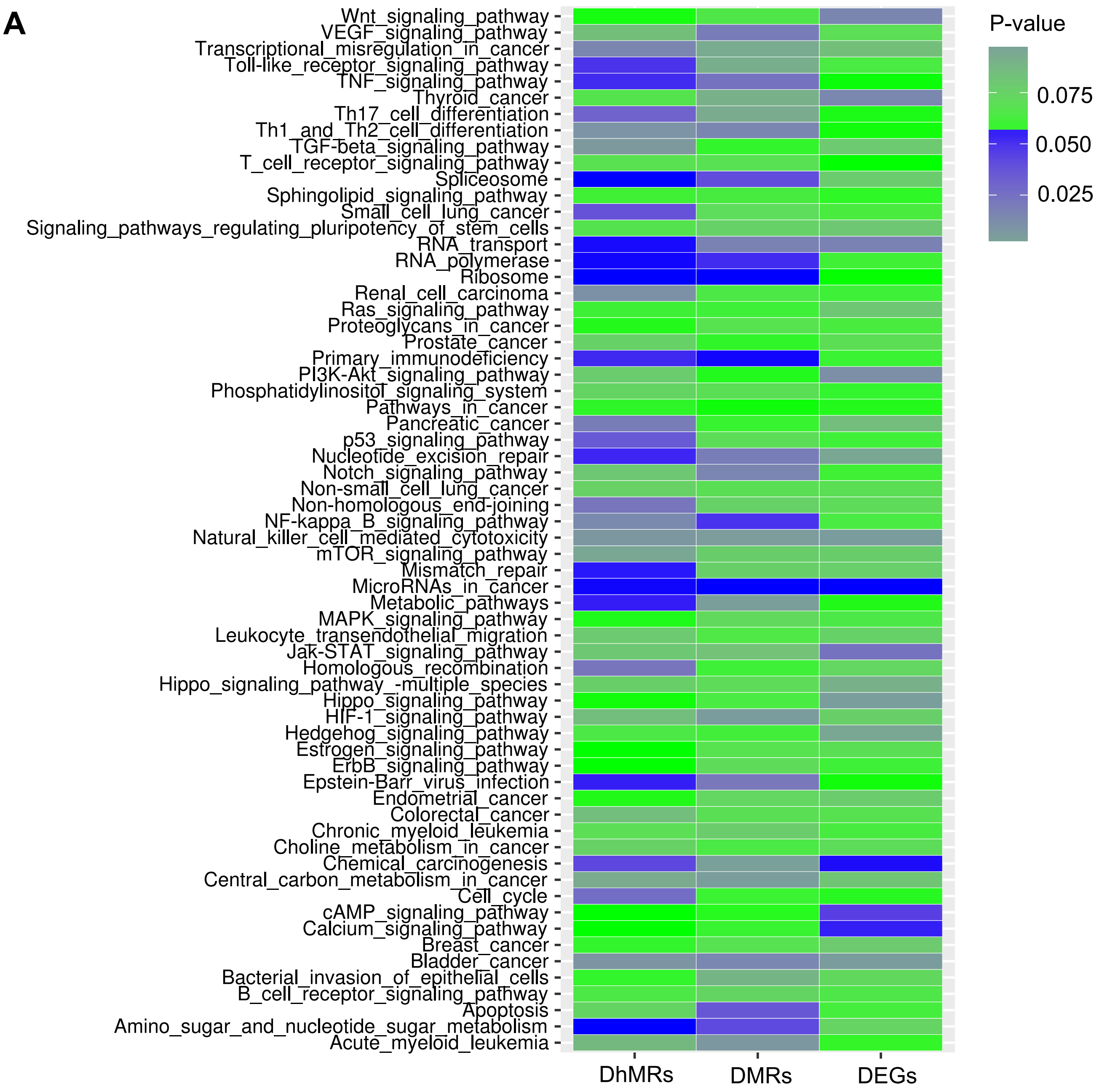

B

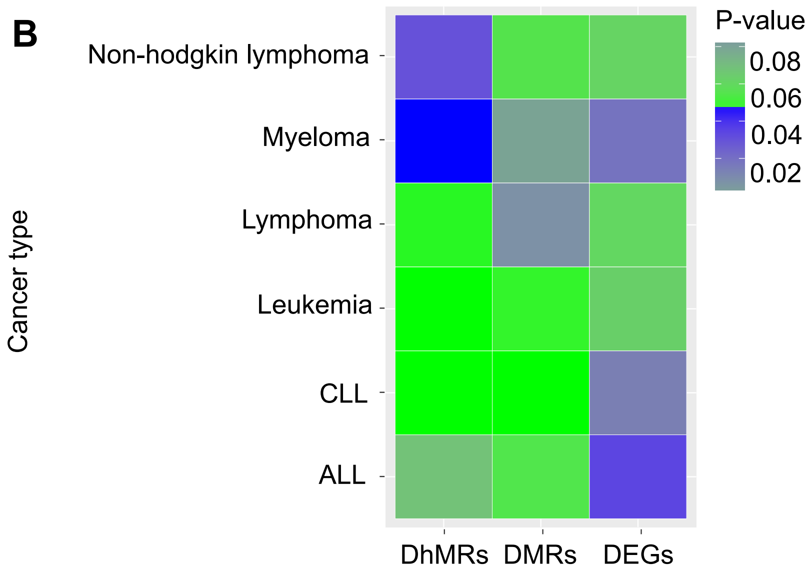

LIST OF ALL THE SUPPLEMENTARY DATA FILES:

Supplementary data file 1

Supplementary data 1A: Clinical and molecular information on CLL patient samples used in this study.

Supplementary data 1B: Summary of obtained reads from CLL samples and normal B cell control samples used in this study.

Supplementary data file 2

Supplementary data 2A: CLL_common_DhMRs from total CLL samples vs Normal sorted B cell sample comparison.

Supplementary data 2B CLL_hyper_DhMRs from total CLL samples vs Normal sorted B cell sample comparison.

Supplementary data 2C: CLL_hypo DhMRs from total CLL samples vs Normal sorted B cell sample comparison.

Supplementary data 2D: CLL_common_DhMRs from IGHV mutated CLL samples vs Normal memory B cell sample comparison.

Supplementary data 2E: CLL_hyper_DhMRs from IGHV mutated CLL samples vs Normal memory B cell sample comparison.

Supplementary data 2F: CLL_hypo_DhMRs from IGHV mutated CLL samples vs Normal memory B cell sample comparison.

Supplementary data 2G: CLL_common_DhMRs from IGHV unmutated CLL samples vs Normal Naive B cell sample comparison.

Supplementary data 2H: CLL_hyper_DhMRs from IGHV unmutated CLL samples vs Normal Naive B cell sample comparison.

Supplementary data 2I: CLL_hypo_DhMRs from IGHV unmutated CLL samples vs Normal Naive B cell sample comparison.

Supplementary data file 3

Supplementary data 3A: CLL_common_DMRs from total CLL samples vs Normal sorted B cell sample comparison.

Supplementary data 3B CLL_hyper_DMRs from total CLL samples vs Normal sorted B cell sample comparison.

Supplementary data 3C: CLL_hypo DMRs from total CLL samples vs Normal sorted B cell sample comparison.

Supplementary data 3D: CLL_common_DMRs from IGHV mutated CLL samples vs Normal memory B cell sample comparison.

Supplementary data 3E: CLL_hyper_DMRs from IGHV mutated CLL samples vs Normal memory B cell sample comparison.

Supplementary data 3F: CLL_hypo_DMRs from IGHV mutated CLL samples vs Normal memory B cell sample comparison.

Supplementary data 3G: CLL_common_DMRs from IGHV unmutated CLL samples vs Normal Naive B cell sample comparison.

Supplementary data 3H: CLL_hyper_DMRs from IGHV unmutated CLL samples vs Normal Naive B cell sample comparison.

Supplementary data 3I: CLL_hypo_DMRs from IGHV unmutated CLL samples vs Normal Naive B cell sample comparison.

Supplementary data file 4

Supplementary data 4A: CLL DEGs from total CLL samples vs normal sorted B cell sample comparison.

Supplementary data 4B: CLL DEGs from IGHV mutated CLL samples vs normal memory B cell sample comparison.

Supplementary data 4C: CLL DEGs from IGHV unmutated CLL samples vs normal Naive B cell sample comparison.

Supplementary data file 5

Supplementary data 5A: REACTOME pathway enrichments of CLL_hyper_DhMRs for total CLL samples vs normal B cells.

Supplementary data 5B: REACTOME pathway enrichments of CLL_hypo_DhMRs for total CLL samples vs normal B cells

Supplementary data 5C: GO Biological pathway enrichments of CLL_hyper_DhMRs for total CLL samples vs normal B cells

Supplementary data 5D: GO Biological pathway enrichments of CLL_hypo_DhMRs for total CLL samples vs normal B cells

Supplementary data 5E: List of total CLL active promoters based on H3K27ac and H3K4me1 peaks

Supplementary data 5F: List of total CLL active enhancers based on H3K27ac peaks

Supplementary data 5G: List of total CLL super enhancers based on H3K27ac peaks

<u>Supplementary data file 6</u>

Supplementary data 6A: Mass spectrometry data of CLL samples and normal B cell subtypes.

Supplementary data 6B: Primer sequences used for validating 5-hmC levels of selected genes from CLL hDMRs
